# Rapid discovery of chemical constituents and absorbed components in rat serum after oral administration of Fuzi-Lizhong pill based on high-throughput HPLC-Q-TOF/MS analysis

**DOI:** 10.1186/s13020-019-0227-z

**Published:** 2019-03-01

**Authors:** Zhen Zhang, Maoyuan Jiang, Xinyi Wei, Jinfeng Shi, Zhao Geng, Shasha Yang, Chaomei Fu, Li Guo

**Affiliations:** 1 The Ministry of Education Key Laboratory of Standardization of Chinese Herbal Medicine, State Key Laboratory Breeding Base of Systematic Research, Development and Utilization of Chinese Medicine Resources, College of Pharmacy, Chengdu University of Traditional Chinese Medicine, Chengdu, 611137 China; 2 Sichuan Institute for Food and Drug Control, Chengdu, 611137 China

**Keywords:** Fuzi-Lizhong pill, Chemical constituents, Bioactive compounds, Metabolites, Traditional Chinese herbal medicine, High-performance liquid chromatography–electrospray ionization/quadrupole-time-of-flight high definition mass spectrometry

## Abstract

**Background:**

Fuzi-Lizhong pill (FZLZP), which was first recorded in the Classic–“Taiping Huimin Heji Ju Fang” of the Song Dynasty, has been widely used to treat gastrointestinal disease in clinic for thousands of years in China. However, an in-depth understanding of the chemical constituents of FZLZP and its potential bioactive constituents is lacking.

**Methods:**

A simple, sensitive and selective method of high-performance liquid chromatography coupled with quadrupole-time-of-flight high-definition mass spectrometry (HPLC-Q-TOF/MS) and automated data analysis (Agilent MassHunter Qualitative Analysis B.06.00 Workstation Software) was developed to simultaneously identify the chemical constituents of FZLZP and the absorbed prototypes as well as the metabolites in rat serum after the oral administration of FZLZP.

**Results:**

Sixty-seven compounds, including alkaloids, flavonoids, triterpenes, gingerols, phenylpropanoids and volatile oil, in the FZLZP extract were tentatively characterized by comparing the retention time and mass spectrometry data and retrieving the reference literatures. Additionally, 23 prototype compounds and 3 metabolites in the rat serum samples were identified after oral administration of FZLZP, which might be the potential active components in vivo. In addition, the absorption of alkaloids decreased when *Aconitum carmichaeli* Debx. was in the form of combined application as a prescription compared to when it was in the form of herb powder.

**Conclusions:**

Herein, the chemical constituent in vitro and the absorbed compounds in the serum of a traditional Chinese formula, Fuzi-Lizhong pill, were fully characterized using a rapid and comprehensive analysis approach based on high-performance liquid chromatography combined with quadrupole time-of-flight mass spectrometry coupled to MassHunter Qualitative Analysis software data processing approach. The results provide helpful chemical information on FZLZP for further pharmacology and active mechanism research. In view of the bioactive constitutes that basically were derived from these absorbed compounds in vivo, this work could provide a useful strategy to explore the bioactive substances of traditional Chinese medicine.

**Electronic supplementary material:**

The online version of this article (10.1186/s13020-019-0227-z) contains supplementary material, which is available to authorized users.

## Background

Fuzi-Lizhong pill (FZLZP) is a popular Traditional Chinese medicine pill that was originally described in the Classic “Taiping Huimin Heji Ju F/ang” of the Song Dynasty (year 1102 by the Western calendar). It is composed of five herbal medicines, including *Aconitum carmichaeli* Debx. (Fuzi), *Codonopsis pilosula* (Franch.) Nannf. (Dangshen), *Atractylodes macrocephala* Koidz. (Baizhu), *Glycyrrhiza uralensis* Fisch. (Gancao) and *Zingiber officinale* Rosc. (Ganjiang). FZLZP is famous for warming the middle-jiao and tonifying the spleen and is used to treat spleen yang deficiency syndrome including enteritis, chronic diarrhoea, irritable bowel syndrome, abdominal pain, vomiting and spasm, peripheral chill, etc. [1–7]. Modern pharmacological research shows that FZLZP possesses a variety of pharmacological activities, including an increase in adaptive thermogenesis, pain relief, anti-inflammation, and spasmolytic benefits [8–15]. Although pharmacological activities of FZLZP have been extensively studied, very little is known about its systematic chemical constituents, and the bioactive compounds that account for its therapeutic effects remain unclear.

In our previous research, we focused on the dissolution behaviour of FZLZP in vitro and the results showed that some constituents in *Aconitum carmichaeli Debx.* and *Glycyrrhiza uralensis* Fisch., such as benzoylaconine, liquiritin and glycyrrhizic acid, were dissolved well in vitro [16–18]. While FZLZP has the so-called active ingredients, there are no empirical data to prove their effectiveness as bioactive compounds. According to the theory of serum pharmacochemistry, while there are multiple components in herbs, only compounds that are absorbed into the blood have the possibility of showing pharmacological bioactivities [19–24]. Therefore, simultaneous identification of systematic chemical constituents in vitro and potential active components in the blood of FZLZP are indispensable.

It was reported that the main components in *Aconitum carmichaeli Debx.* are monoester diterpenoid alkaloids (MDAs) and diester diterpenoid alkaloids (DDAs), which are toxicity and efficacy compounds and should be highly concerned [21]. Due to the toxicity, Fuzi is usually used in combination with other herbs as a prescription. Some researcher considered the combination to cause the reduction of the absorption of toxic compounds [21, 25]. As a typical combination, however, there are no detailed studies of this mechanism and the compound variations of FZLZP. The strategy of serum thermochemistry can provide us the accurate qualitative and the preliminary quantitative information for exploring the quantitative change of alkaloids and toxicity reducing mechanism.

Currently, LC–MS is widely applied for the analysis of herbal constituents in vitro and in vivo because of its superior sensitivity, selectivity and ability to conclusively identify the compounds [26–29]. In this study, an approach of high-performance liquid chromatography (HPLC) quadrupole time-of-flight mass spectrometry (QTOF-MS) based on serum pharmacochemistry was developed to identify the phytochemical constituents of FZLZP and multiple absorbed components in rat serum.

## Methods

The Minimum Standards of Reporting Checklist contains details of the experimental design, and statistics, and resources used in this study (Additional file 1).

### Chemicals and materials

Nine reference compounds were obtained from Sichuan Victor Biological Technology Co. Ltd. (Chengdu China). HPLC grade Ethanol, formic acid and methanol were obtained from Fisher (ThermoFisher Scientific Inc, Waltham, MA, USA). Deionised water (18 MΩ) was prepared by distilled water through a Milli-Q system (Millipore, Milford, MA, USA). Fuzi (No. 1703003), Dangshen (No. 1705003), Baizhu (No. 1704088), Ganjiang (No. 1703060) and Gancao (No. 1703034) were purchased from Sichuan Neautus Traditional Chinese Medicine Co., Ltd. (Chengdu China) and were authenticated by Prof. Jin Pei, Department of Pharmacognosy of Chengdu University of Chinese Medicine.

### Preparation of FZLZP

Fuzi, Ganjiang, Dangshen, Baizhu and Gancao were ground into fine powers and weighed according to the instructions recorded in Chinese Pharmacopoeia (2015 edition) and mixed well. Honey was heated at 116–118 °C until bright yellow uniform bubbles appeared on the surface and the honey became sticky. Mixed power and thermal refined honey were mixed at a ratio of 1:0.8 and were made into FZLZP (there is 0.153 kg crude aconite for every 1 kg FZLZP).

### Preparation of FZLZP extract samples for LC/MS analysis

FZLZP (1.5 g) was weighed and reflux-extracted with 50 mL 70% ethanol for 1 h. Then, the filtered supernatant sample was rotary evaporated at 40 °C to a concentration of 15 mL, and was centrifuged at 5000 revolutions/min (rpm) for 5 min. The solution was filtered through a 0.22-μm membrane for further analysis.

### Animal handling and serum sample preparation

Eighteen male Sprague–Dawley rats (200 ± 20 g) were obtained from the Sichuan Dashuo Biotechnology Co., Ltd. and were randomly divided into three groups of 6 rats each (group A, FZLZP group for dosed rat serum; group B, Fuzi powder (FZP) group for dosed rat serum; group C, control group for blank rat serum). The animal facilities and protocols conformed to the Care and Use of Laboratory Animals published by the National Institutes of Health. The experiment was approved by the ethical committee of Chengdu University of TCM (No.20161105). The rats were housed in an animal room with a controlled environment (20–25 °C, 65–69% relative humidity, 12 h dark–light cycle), and were given water and fed normal food for 1 week before the experiment. All animals were fasted overnight before the experiments and had free access to water.

The FZLZP was dissolved in 0.5% CMC-Na and were grinded to prepare the FZLZP suspension (150 mg crude drug/mL). Fuzi powder was dissolved in 0.5% CMC-Na to prepare the FZPsuspension (23 mg crude drug/mL, the concentration of FuZi was calculated by the ratio in FZLZP). Group A was intragastric administration 1.5 g/kg body weight of FZLZP suspension for 3 days. Group B was intragastric administration 0.23 g/kg body weight of FZP suspension for 3 days. Group C was intragastric administration with an equivalent volume of 0.5% CMC-Na. Blood samples were collected from the abdominal aorta 45 min after oral administration on the 3rd day and were placed at room temperature for 1 h until solidification. Then, samples were centrifuged at 3000 rpm for 10 min at 4 °C. All samples were stored at − 80 °C until analysis. Three times methanol was added to the 2 mL serum samples, vortexed and then, centrifuged at 12,000 rpm for 20 min. The supernatant was dried with nitrogen gas. The residue was redissolved in 50 μL methanol, vortexed and then, centrifuged at 12,000 rpm for 20 min, and the filtrate was used as the LC/MS sample. 10 µL aliquot was injected for HPLC/MS analysis.

### HPLC-QTOF-MS analysis condition

Chromatographic analysis was performed in an Agilent 1290 HPLC system controlled with MassHunter Workstation Software (V B.05.00, Agilent Technologies Inc, Santa Clara, CA, USA). Samples were separated on an Agilent HC-C_18_ column (4.6 × 250 mm, 5.0 μm, Agilent Technologies Inc.) held at 35 °C and the flow rate was 1.0 mL/min with the injection volume of 10 μL. The mobile phase consisted of 0.1% formic acid–water (v/v, A) and methanol (B). The optimal gradient elution programme was as follows: 0–15 min, 95–70% A; 15–30 min, 70–48% A; 30–45 min, 48–25% A; 45–48 min, 25–15% A; 48–55 min, 15–2% A; and 55–65 min, 2–2% A.

### Mass spectrometry conditions

Mass spectrometry was performed using an Agilent 6540 QTOF–MS (Agilent Corp., USA) equipped with a Dual AJS electrospray ionization (ESI) source, and the following operating parameters were used: positive mode, drying gas (nitrogen, N_2_); flow rate, 8.0 L/min; gas temperature, 325 °C; nebulizer, 40 psig; sheath gas temperature, 350 °C; sheath gas flow, 11 L/min; capillary voltage, 4000 V; skimmer, 65 V; OCT 1 RF Vpp, 750 V; fragmentor, 110 V. The sample collision energy was set at 10, 20, 30 and 40 V. All the operations, acquisition, and analyses of data were controlled by Agilent LCMS-QTOF Mass Hunter Acquisition Software Ver. B.06.00 (Agilent Technologies Inc.) and operated under Mass Hunter Workstation Software Version B.06.00 (Agilent Technologies Inc.).

### Establishment of FZLZP database

By searching databases, such as PubMed of the US National Library Medicine and the National Institutes of Health, SciFinder Scholar of American Chemical Society and the Chinese National Knowledge Infrastructure (CNKI) of Tsinghua University, all components reported in the literature on *Aconitum carmichaeli* Debx., *Codonopsis pilosula* (Franch.) Nannf., *Atractylodes macrocephala* Koidz., *Glycyrrhiza uralensis* Fisch. and *Zingiber officinale* Rosc. were summarized in an Agilent PCDL software Ver. B.06.00 (Agilent Technologies Inc.) to establish a database, which includes the name, molecular formula, chemical structure and literatures of each published known compound.

## Results

### Characterization of chemical constituents from FZLZP

**Fig. 1:**
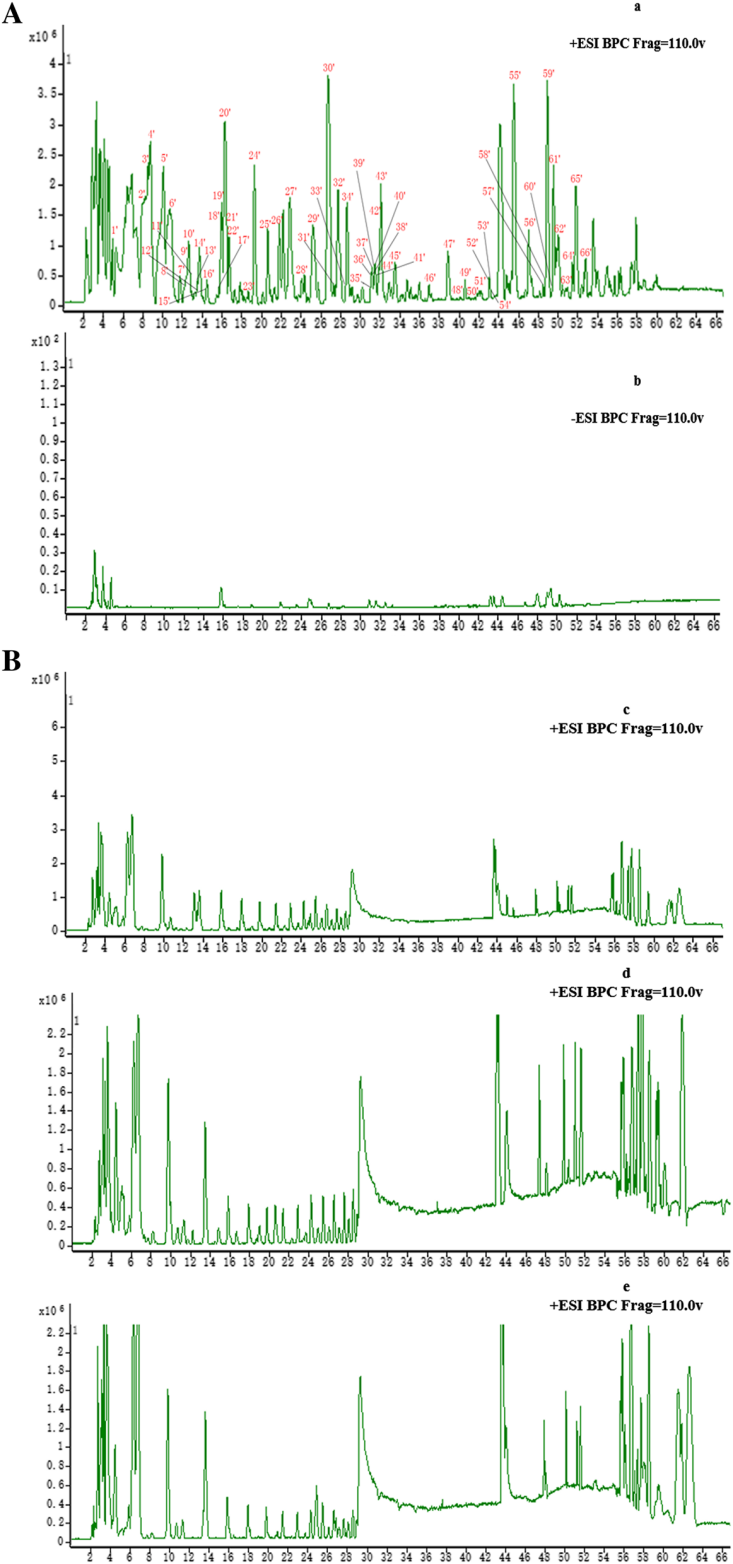
The HPLC-ESI/QTOF/MS BPC chromatograms (**A** FZLZP extract samples: **a** in positive mode, **b** in negative mode; **B** Serum samples: **c** controlled serum in positive mode, **d** dosed FZLZP serum in positive mode, **e** dosed FZP serum in positive mode.)

Using the optimal conditions described above, all information on the MS data that was obtained from the robust HPLC-TOF-MS analysis, indicated the retention time and precise molecular mass and provided the MS/MS data. The protonated molecular weights of all target compounds were calculated within an error of 5 ppm. The base peak chromatogram (BPC) of the FZLZP extract sample in positive and negative ion modes are shown in Fig. 1A, and the data were processed by the Agilent MassHunter Qualitative Analysis B.06.00 Workstation Software with the “find compounds by molecular formula” tool. A total of 73 peaks were obtained, and 67 compounds were identified or tentatively characterized by comparing the *t*_*R*_ values and the MS fragment characteristics of the compounds.

**Fig. 2:**
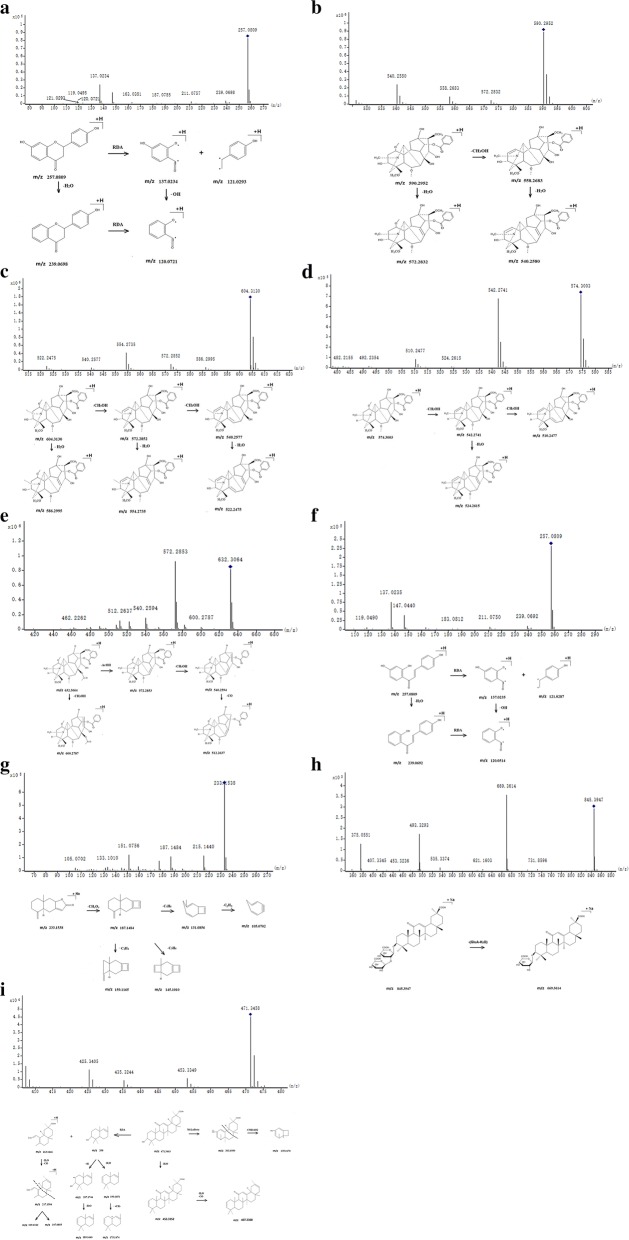
The mass fragment and fragmentation pathway of **a** Liquiritigenin, **b** Benzoylmesaconine, **c** Benzoylaconine, **d** Benzoylhypaconine, **e** mesaconitine, **f** Isoliquiritigenin, **g** Atractylenolide II, **h** Glycyrrhizic acid, **i** Glycyrrhetinic acid

The reference standards are summarized in Table 1 and their fragmentation mechanism are proposed in Fig. 2. The compounds in FZLZP which are identified by the reference standards are summarized and marked in Table 2. For example, reference standards (RS) 1 liquiritigenin in Table 1 were detected in the positive ion mode at the Rt in 24.843 min with the *m/z* of 257.0809 (C_15_H_13_O_4_). Its MS/MS data were shown as *m/z* of 239.0698[M + H–H_2_O]^+^, 137.0234 [C_7_H_4_O_3_ + H]^+^, 121.0293[C_8_H_8_O + H]^+^ and 120.0721 [C_7_H_4_O_3_ + H–OH]^+^. And the compound 29 in Table 2 were detected in the positive ion mode at the Rt in 24.785 min with the *m/z* of 257.0819 (C_15_H_13_O_4_), 239.0707[M + H–H_2_O]^+^ and 137.0235 [C_7_H_4_O_3_ + H]^+^. Then compound 29 were characterized as liquiritigenin. Similar to the identification process above, among 67 compounds, 9 compounds were identified as benzoylaconine, benzoylmesaconine, benzoylhypaconine, mesaconitine, liquiritigenin, isoliquiritigenin, glycyrrhizic acid, glycyrrhetinic acid and atractylenolide II. The MS data of the (+) ESI–MS spectra are shown in Table 2.

**Table 1:** Retention time, m/z values of ions of reference standards

Peak no.	Rt (min)	Systematic name	Molecular formula	[M + H]^+^		[M + Na]^+^		Fragmentations (m/z)
				Measured mass (m/z)	Error (ppm)	Measured mass (m/z)	Error (ppm)	
1	24.843	Liquiritigenin	C_15_H_12_O_4_	257.0809	0.3890			257.0809[M + H]^+^, 239.0698[M + H–H_2_O]^+^, 137.0234 [C_7_H_4_O_3_ + H]^+^, 121.0293 [C_8_H_8_O + H]^+^, 120.0721 [C_7_H_4_O_3_ + H–OH]^+^
2	27.507	Benzoylmesaconine	C_31_H_43_NO_10_	590.2952	− 1.3553	–	–	590.2952[M + H]^+^, 572.2832[M + H–H_2_O]^+^, 558.2683[M + H-CH_3_OH]^+^, 540.2580[M + H–CH_3_OH–H_2_O]^+^
3	28.228	Benzoylaconine	C_32_H_45_NO_10_	604.3130	2.3167	–	–	604.3130[M + H]^+^, 586.2995[M + H–H_2_O]^+^, 572.2852[M + H–CH_3_OH]^+^ 554.2735[M + H–2H_2_O]^+^, 540.2577[M + H–CH_3_OH]^+^, 522.2475[M + H–2CH_3_OH–H_2_O]^+^
4	29.152	Benzoylhypaconine	C_31_H_43_NO_9_	574.3003	− 1.3930	–	–	574.3003[M + H] + , 542.2741[M + H–CH_3_OH]^+^, 524.2615[M + H–CH_3_OH–H_2_O]^+^, 510.2477[M + H–2CH_3_OH]^+^
5	31.663	Mesaconitine	C_33_H_45_NO_11_	632.3064	− 0.1582	–	–	632.3064[M + H]^+^, 600.2787[M + H–CH_3_OH]^+^, 572.2853[M + H–AcOH]^+^, 540.2594[M + H–AcOH–CH_3_OH]^+^, 512.2637[M + H–AcOH–CH_3_OH–CO]^+^,
6	39.648	Isoliquiritigenin	C_15_H_12_O_4_	257.0809	0.3890			257.0809[M + H]^+^, 239.0692[M + H–H_2_O]^+^, 137.0235[C_7_H_4_O_3_ + H]^+^, 121.0287 [C_8_H_8_O + H]^+^, 120.0514 [C_7_H_4_O_3_ + H–OH]^+^
7	48.854	Atractylenolide II	C_15_H_20_O_2_	233.1538	0.8578			233.1538[M + Na]^+^, 215.1440[M + Na–H_2_O]^+^, 187.1484[M + Na–CH_2_O_2_]^+^, 159.1165[M + Na–CH_2_O_2_–C_2_H_4_]^+^, 145.101 [M + Na–CH_2_O_2_–C_3_H_6_]^+^ 131.0856[M + Na–CH_2_O_2_–C_4_H_8_]^+^, 105.0702[M + Na–CH_2_O_2_–C_4_H_8_–C_2_H_2_]^+^,
8	49.134	Glycyrrhizic acid	C_42_H_62_O_16_			845.3947	2.0109	845.3947[M + Na]^+^, 669.3614[M + Na–(GluA–H_2_O)]^+^
9	55.125	Glycyrrhetinic acid	C_30_H_46_O_4_	471.3458	− 2.3337			471.3458[M + H]^+^, 453.3349[M + H–H_2_O]^+^, 435.3244[M + H-2H_2_O]^+^

**Table 2:** Identification information of constituents in vitro of FZLZP by HPLC-ESI/QTOF/MS

Peak no.	Rt (min)	Systematic name	Molecular formula	Molecular weight	[M + H]^+^		[M + Na]^+^		Fragmentations (m/z)	Source
					Measured mass (m/z)	Error (ppm)	Measured mass (m/z)	Error (ppm)		
1	5.091	l-Pyroglutamic acid	C_5_H_7_NO_3_	129.0426	130.0505	4.6136			130.0505[M + H]^+^, 112.0123[M + H–H_2_O]^+^, 84.0449[M + H–HCOOH]^+^	Dangshen
2	8.051	Codonopsine	C_14_H_21_NO_4_	267.1471	268.1543	0			268.1543[M + H]^+^, 250.1451[M + H–H_2_O]^+^, 205.0863[M + H–2CH_3_OH]^+^	Dangshen
3	9.229	5-hydroxymethyfurfural	C_6_H_6_O_3_	126.0317	127.0394	3.1486			127.0394[M + H]^+^, 109.0291[M + H–H_2_O]^+^	Dangshen
4	9.398	Karakolidine	C_22_H_35_NO_5_	393.2515	394.2590	0.5072			394.2590[M + H]^+^, 376.2489[M + H–H_2_O]^+^, 358.2371[M + H–2H_2_O]^+^	Fuzi
5	10.142	Phenylalanine	C_9_H_11_NO_2_	165.0790	166.0872	5.4188			166.0872[M + H]^+^, 120.0817[M + H–HCOOH]^+^	Dangshen
6	11.288	Senbusine A	C_23_H_37_NO_6_	423.2621	424.2696	0.4713			424.2696[M + H]^+^, 406.2579 [M + H–H_2_O]^+^	Fuzi
7	11.407	9-OH-senbusine A	C_23_H_37_NO_7_	439.2570	440.2635	− 1.8170			440.2635[M + H]^+^, 422.2531[M + H–H_2_O]^+^, 408.2318[M + H–CH_3_OH]^+^	Fuzi
8	12.042	16-β-hydroxycardiopetaline	C_21_H_33_NO_4_	363.2410	364.2480	− 0.5490			364.2480[M + H]^+^, 346.2372[M + H–H_2_O]^+^, 328.2273[M + H–2H_2_O]^+^	Fuzi
9	12.389	Mesaconine	C_24_H_39_NO_9_	485.2625	486.2697	− 0.2056			486.2697 M + H]^+^, 468.2573[M + H–H_2_O]^+^, 436.2323[M + H–H_2_O–CH_3_OH]	Fuzi
10	12.578	Songorine	C_22_H_31_NO_3_	357.2304	358.2382	1.3957			358.2382[M + H]^+^, 340.2267[M + H–H_2_O]^+^	Fuzi
11	12.908	Karakoline	C_22_H_35_NO_4_	377.2566	378.2639	0			378.2639[M + H]^+^, 360.2533[M + H–H_2_O]^+^	Fuzi
12	13.081	Isotalatizidine	C_23_H_37_NO_5_	407.2672	408.2743	− 0.2449			408.2743[M + H]^+^, 390.2630[M + H–H_2_O]^+^, 372.2517[M + H–2H_2_O]^+^, 358.2374[M + H–H_2_O–CH_3_OH]^+^	Fuzi
13	13.109	Senbusine B	C_23_H_37_NO_6_	423.2621	424.2707	3.0640			424.2707[M + H]^+^, 406.2584 [M + H–H_2_O]^+^	Fuzi
14	13.937	14-Acetylkarakoline	C_24_H_37_NO_5_	419.2672	420.2750	1.4276			420.2750[M + H]^+^, 402.1695[M + H–H_2_O]^+^, 356.1122[M + H–H_2_O–2CH_3_OH]^+^,	Fuzi
15	14.091	Aconine	C_25_H_41_NO_9_	499.2781	500.2850	− 0.7995			500.2850[M + H]^+^, 482.2741[M + H–H_2_O]^+^, 468.2564[M + H–CH_3_OH]^+^, 450.2478[M + H–H_2_O–CH_3_OH]^+^, 436.2309[M + H–2CH_3_OH]^+^, 418.2209[M + H–H_2_O–2CH_3_OH]^+^	Fuzi
16	14.380	Hetisine	C_20_H_27_NO_3_	329.1991	330.2064	0			330.2064[M + H]^+^, 312.1951[M + H–H_2_O]^+^	Fuzi
17	15.319	Hypaconine	C_24_H_39_NO_8_	469.2676	470.2744	− 0.8506			470.2744[M + H]^+^, 453.2301[M + H–OH]^+^, 438.2474[M + H–CH_3_OH]^+^, 406.2212[M + H–2CH_3_OH]^+^, 374.1941[M + H–3CH_3_OH]^+^	Fuzi
18	15.810	Fuzitine	C_20_H_23_NO_4_	341.1627	342.1697	− 0.8767			342.1697[M + H]^+^, 324.1026[M + H–H_2_O]^+^	Fuzi
19	16.070	Fuziline	C_24_H_39_NO_7_	453.2727	454.2800	0.2201			454.2800[M + H]^+^, 436.2677[M + H–H_2_O]^+^, 418.2583[M + H–2H_2_O]^+^, 404.2443[M + H–H_2_O–CH_3_OH]^+^, 386.2295[M + H–2H_2_O–CH_3_OH]^+^, 354.2069[M + H–2H_2_O–2CH_3_OH]^+^	Fuzi
20	16.248	Tau-cadinol	C_15_H_26_O	222.1984	245.1852	− 9.7884			245.1852[M + H]^+^, 213.0195[M + H–CH_3_OH]^+^, 199.1252[M + H–CH_3_OH–CH_3_]^+^, 184.9885[M + H–CH_3_OH–2CH_3_]^+^, 169.0055[M + H–CH_3_OH–3CH_3_]^+^,	Ganjiang
21	16.573	Neoline	C_24_H_39_NO_6_	437.2777	438.2848	− 0.4563			438.2848 M + H]^+^, 420.2756[M + H–H_2_O]^+^, 388.2478[M + H–H_2_O–CH_3_OH]^+^, 370.2365[M + H–2H_2_O–CH_3_OH]^+^, 356.2213[M + H–H_2_O–2CH_3_OH]^+^	Fuzi
22	16.743	Talatisamine	C_24_H_39_NO_5_	421.2828	422.2899	0.4736			422.2899[M + H]^+^, 390.2621[M + H–CH_3_OH]^+^, 358.2349[M + H–2CH_3_OH]^+^	Fuzi
23	18.651	Chasmanine	C_25_H_41_NO_6_	451.2934	452.3008	02210			452.3008[M + H]^+^, 420.2737[M + H–CH_3_OH]^+^	Fuzi
24	19.739	Geranial	C_10_H_16_O	152.1201	153.1275	0.6530			153.1275[M + H]^+^, 135.1162[M + H–H_2_O]^+^, 125.0940[M + H–CO]^+^	Ganjiang
25	20.390	14-Acetyltalatizamine	C_26_H_41_NO_6_	463.2934	464.3014	1.5076			464.3014[M + H]^+^, 432.2753[M + H–CH_3_OH]^+^, 414.2645[M + H–CH_3_OH–H_2_O]^+^, 400.2486[M + H–2CH_3_OH]^+^, 372.2522[M + H–CH_3_OH–AcOH]^+^	Fuzi
26	21.828	7-hydroxycoumarin	C_9_H_6_O_3_	162.0317	163.0395	3.0667			163.0395[M + H]^+^, 145.0627[M + H–H_2_O]^+^	Baizhu
27	23.891	Schaftoside	C_26_H_28_O_14_	564.1479	565.1542	− 1.7694			565.1542[M + H]^+^, 547.1434[M + H–H_2_O]^+^, 529.1303[M + H–2H_2_O]^+^, 511.1220[M + H–3H_2_O]^+^	Gancao
28	24.041	Scopoletin	C_10_H_8_O_4_	192.0423	193.0500	2.5900			193.0500[M + H]^+^, 161.0603[M + H–CH_3_OH]^+^	Baizhu
29^#^	24.785	Liquiritigenin	C_15_H_12_O_4_	256.0736	257.0819	4.2788			257.0819[M + H]^+^, 239.0707[M + H–H_2_O]^+^, 137.0235[C_7_H_4_O_3_ + H]^+^, 121.0280[C_8_H_8_O + H]^+^, 120.0525 [C_7_H_4_O_3_ + H–OH]^+^	Gancao
30^#^	27.065	Benzoylmesaconine	C_31_H_43_NO_10_	589.2887	590.2959	− 0.1694	–	–	590.2959[M + H]^+^, 572.2826[M + H–H_2_O]^+^, 558.2663[M + H–CH_3_OH]^+^ 540.2573[M + H–CH_3_OH–H_2_O]^+^	Fuzi
31	27.325	Isoviolanthin	C_27_H_30_O_14_	578.1636	579.1700	− 1.3812			579.1700[M + H]^+^, 561.1588[M + H–H_2_O]^+^, 543.1485[M + H–2H_2_O]^+^, 525.1382[M + H–3H_2_O]^+^	Gancao
32^#^	27.614	Benzoylaconine	C_32_H_45_NO_10_	603.3043	604.3114	− 0.3309			604.3114[M + H]^+^, 587.2801[M + H–OH]^+^, 554.2711[M + H–2CH_3_OH]^+^	Fuzi
33^#^	28.595	Benzoylhypaconine	C_31_H_43_NO_9_	573.2938	574.3011	0			574.3011[M + H]^+^, 542.2745[M + H–CH_3_OH]^+^,,510.2457[M + H–2CH_3_OH]^+^	Fuzi
34	28.748	Lobetyolinin	C_26_H_38_O_13_	558.2312			581.2203	− 0.3441	581.2203[M + Na]^+^, 419.1709[M + Na–C_6_H_10_O_5_]^+^	Dangshen
35	31.019	Liquiritin apioside or Isoliquiritin apioside	C_26_H_30_O_13_	550.1686	551.1751	− 1.4514			551.1751[M + H]^+^, 419.1333[M + H–(Apiose–H_2_O)]^+^, 257.0830[M + H–(Apiose–H_2_O)–(Glc–H_2_O)]^+^	Gancao
36^#^	31.163	Mesaconitine	C_33_H_45_NO_11_	631.2993	632.3067	0.3163	–	–	632.3067[M + H]^+^, 614.1110[M + H–H_2_O]^+^, 600.2748[M + H–CH_3_OH]^+^, 572.2834[M + H–AcOH]^+^	Fuzi
37	31.423	7-methoxy-liquiritin	C_22_H_22_O_9_	430.1264	431.1332	− 1.1597			431.1332[M + H]^+^, 269.0811[M + H–(Glc–H_2_O)]^+^	Gancao
38	31.646	14-Benzoylneoline	C_31_H_43_NO_7_	541.3040	542.3135	4.2411			542.3135[M + H]^+^, 524.3010[M + H–H_2_O]^+^, 510.2731[M + H–CH_3_OH]^+^, 492.2733[M + H–H_2_O–CH_3_OH]^+^	Fuzi
39	31.659	Dehydrated benzoylhypaconine	C_31_H_41_NO_8_	555.2832	556.2906	0.1798			556.2906[M + H]^+^, 524.2647[M + H–CH_3_OH]^+^, 492.2381[M + H–2CH_3_OH]^+^	Fuzi
40	31.683	Liquiritin or Isoliquiritin	C_21_H_22_O_9_	418.1264	419.1335	0.4771			419.1335[M + H]^+^, 257.0811[M + H–(Glc–H_2_O)]^+^	Gancao
41	31.921	Aconifine	C_34_H_47_NO_12_	661.3098	662.3172	0.1509			662.3172[M + H]^+^, 644.3095[M + H–H_2_O]^+^, 626.1346 [M + H–2H_2_O]^+^	Fuzi
42	32.100	Hypaconitine	C_33_H_45_NO_10_	615.3043	616.3116	0			616.3116[M + H]^+^, 584.2843[M + H–CH_3_OH]^+^ 556.2899[M + H–C_2_H_4_O_2_]^+^, 524.2533[M + H–C_2_H_4_O_2_–CH_3_OH]^+^, 496.2678[M + H–C_2_H_4_O_2_–CH_3_OH–CO]^+^	Fuzi
43	32.245	Formononetin	C_16_H_12_O_4_	268.0736	269.0814	2.2298			269.0814[M + H]^+^, 254.0580[M + H–CH_3_]^+^, 237.0536[M + H–CH_3_OH]^+^, 225.0554[M + H–CH_3_–CO]^+^, 213.0908[M + H–C_2_O_2_]^+^, 181.0666[M + H–C_2_O_2_–CH_3_OH]^+^	Gancao
44	32.528	Aconitine	C_34_H_47_NO_11_	645.3149	646.3216	− 0.9283			646.3216[M + H]^+^, 628.3140[M + H–H_2_O]^+^, 596.2849[M + H–H_2_O–CH_3_OH]^+^	Fuzi
45	33.241	Deoxyaconitine	C_34_H_47_NO_10_	629.3200	630.3273	0			630.3273[M + H]^+^, 598.3070[M + H–CH_3_OH]^+^	Fuzi
46	36.853	Echinatin	C_16_H_14_O_4_	270.0892	271.0963	− 0.7377			271.0963[M + H]^+^, 253.0850[M + H–H_2_O]^+^	Gancao
47	38.085	Benzoic acid	C_7_H_6_O_2_	122.0368	123.0447	4.8763			123.0447[M + H]^+^, 77.0379[M + H–HCOOH]^+^	Baizhu
48^#^	39.763	Isoliquiritigenin	C_15_H_12_O_4_	256.0736	257.0814	2.334			257.0814[M + H]^+^, 239.0704[M + H–H_2_O]^+^, 137.0235[C_7_H_4_O_3_ + H]^+^, 121.0277[C_8_H_8_O + H]^+^, 120.0527 [C_7_H_4_O_3_ + H–OH]^+^	Gancao
49	40.720	Glycycoumarin	C_21_H_20_O_6_	368.1260	369.1345	3.2508			369.1345[M + H]^+^, 333.2235[M + H–2H_2_O]^+^, 313.1057 [M + H–C_4_H_8_]^+^,	Gancao
50	41.513	6-gingerdione	C_17_H_24_O_4_	292.1675	293.1736	− 2.7520			293.1736[M + H]^+^, 275.1650[M + H–H_2_O]^+^ 257.1517[M + H–2H_2_O]^+^	Ganjiang
51	42.593	Kumatakenin	C_17_H_14_O_6_	314.0790	315.0859	− 1.2694			315.0859[M + H]^+^, 298.2146[M + H–OH]^+^, 279.0782[M + H–2H_2_O]^+^	Ganjiang
52	43.486	6-gingerol	C_17_H_26_O_4_	294.1831			317.1737	4.4140	317.1771[M + Na]^+^, 299.2546[M + Na-H_2_O]^+^	Ganjiang
53	43.507	Gingerenone-A	C_21_H_24_O_5_	356.1624	357.1710	3.6397			357.1710[M + H]^+^, 339.2718[M + H–H_2_O]^+^, 321.2612[M + H–2H_2_O]^+^	Ganjiang
54	43.544	6-shogaol	C_17_H_24_O_3_	276.1725	277.1795	1.0823			277.1795[M + H]^+^, 259.1694[M + Na–H_2_O]^+^,	Ganjiang
55	45.779	lupiwighteone	C_20_H_18_O_5_	338.1154	339.1239	3.5385			339.1239[M + H]^+^, 321.2818[M + H–H_2_O]^+^	Gancao
56	46.339	Atractylenolide III	C_15_H_20_O_3_	248.1412	245.1485	0			249.1485[M + H]^+^, 231.1389[M + H–H_2_O]^+^, 175.0751[M + H–H_2_O–2CO]^+^, 163.0756[M + H–H_2_O–C_5_H_8_]^+^	Baizhu
57	48.364	Gancaonin L	C_20_H_18_O_6_	354.1103	355.1189	3.6607			355.1189[M + H]^+^, 337.2536[M + H–H_2_O]^+^	Gancao
58	48.398	Licoricesaponin G2	C_42_H_62_O_17_	838.3987	839.4076	1.9061			839.4076[M + H]^+^, 663.3722[M + H–(GluA–H_2_O)]^+^, 469.3308[M + H–2 (GluA–H_2_O)–H_2_O]^+^	Gancao
59^#^	48.887	Atractylenolide II	C_15_H_20_O_2_	232.1463	233.1541	2.1445			233.1541[M + Na]^+^, 187.1485[M + Na–CH_2_O_2_]^+^, 159.0806[M + Na–CH_2_O_2_–C_2_H_4_]^+^, 145.1013 [M + Na–CH_2_O_2_–C_3_H_6_]^+^, 131.0857[M + Na–CH_2_O_2_–C_4_H_8_]^+^, 105.0703[M + Na–CH_2_O_2_–C_4_H_8_–C_2_H_2_]^+^	Baizhu
60^#^	49.296	Glycyrrhizic acid	C_42_H_62_O_16_	822.4038	823.4130	2.3075			823.4130 [M + H]^+^, 647.3793[M + H–(GluA–H_2_O)]^+^	Gancao
61	49.667	Farnesal	C_15_H_24_O	220.1827	221.1907	3.1647			221.1907 M + H]^+^, 192.9740[M + H–CO]^+^	Ganjiang
62^#^	49.841	Glycyrrhetinic acid	C_30_H_46_O_4_	470.3396	471.3488	4.031			471.3488[M + H]^+^, 453.3354[M + H–H_2_O]^+^, 435.3224[M + H–2H_2_O]^+^, 425.3378[M + H–HCOOH]^+^	Gancao
63	50.671	Licorice saponin B2	C_42_H_64_O_15_	808.4245			831.4151	1.6838	831.4151 [M + Na]^+^, 655.3825[M + Na–(GluA–H_2_O)]^+^, 479.3547[M + Na–2 (GluA–H_2_O)]^+^	Gancao
64	51.232	Licoricone	C_22_H_22_O_6_	382.1416	383.1502	3.3929			383.1502[M + H]^+^, 355.1587[M + H–C_2_H_4_]^+^	Gancao
65	51.390	Atractylenolide I	C_15_H_18_O_2_	230.1307	231.1383	1.2979			231.1383[M + H]^+^, 185.1326[M + H–HCOOH]^+^, 157.1012[M + H–HCOOH–C_2_H_4_]^+^, 105.0701 [M + H–HCOOH–2C_2_H_4_–2C]^+^	Baizhu
66	52.950	Neoglycyrol	C_21_H_18_O_6_	366.1103	367.1165	− 0.5447			367.1165[M + H]^+^, 349.2239[M + H–H_2_O]^+^, 335.2389[M + H–CH_3_OH]^+^, 317.2283[M + H–2H_2_O–CH_3_OH]^+^	Gancao
67	54.310	Licorice-saponin J2	C_42_H_64_O_16_	824.4194	825.4286	2.3018			825.4286[M + H]^+^, 649.3906 [M + H–(GluA–H_2_O)]^+^, 455.3537[M + H-2 (GluA–H_2_O)–H_2_O]^+^, 437.3435 [M + H-2 (GluA–H_2_O)–2H_2_O]^+^	Gancao

^#^Indicates compounds identified by comparing with the reference standards

**Fig. 3:**
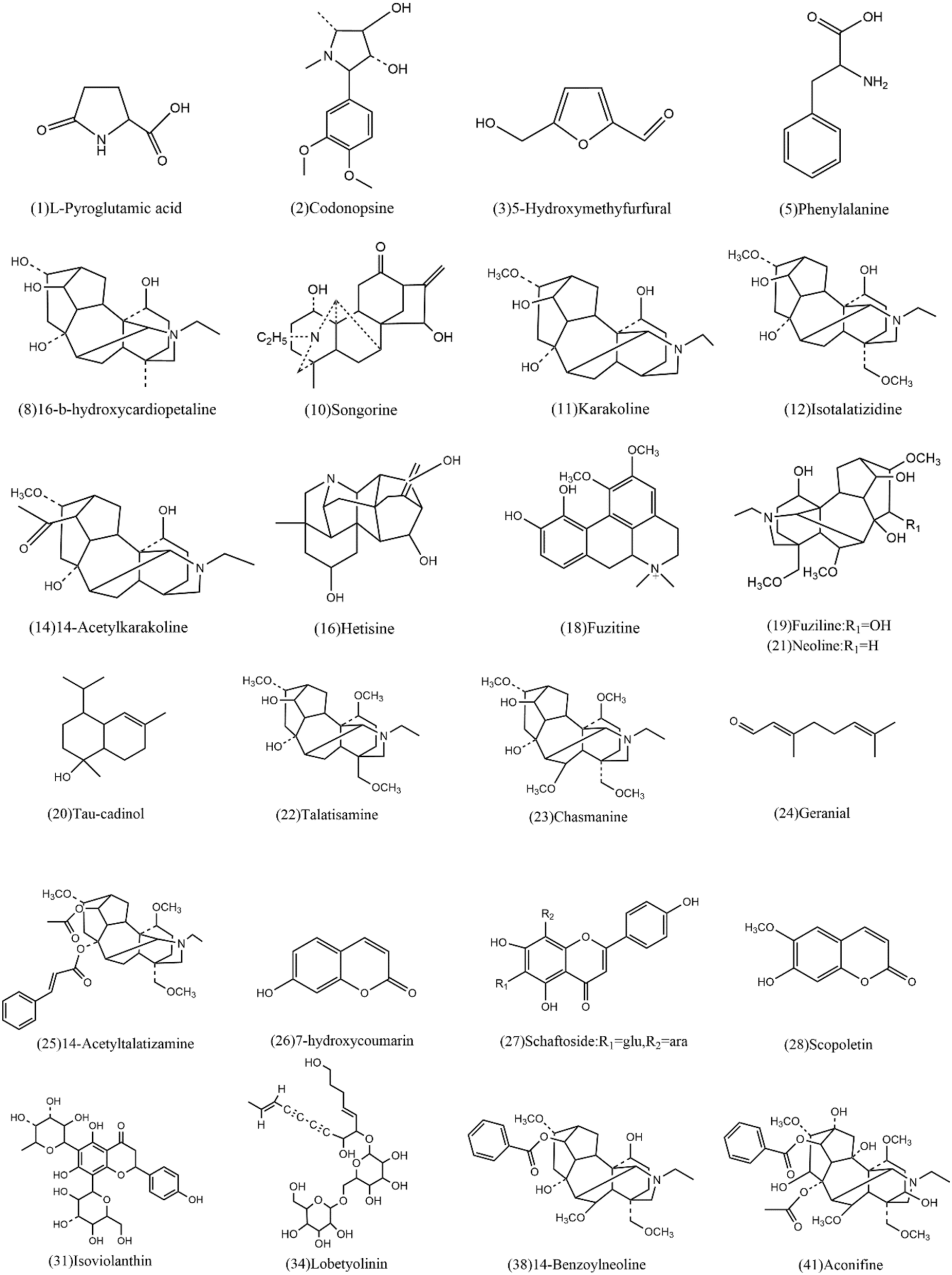
Structures of compounds identified in the extract of Fuzi Lizhong Pill

**Fig. 4:**
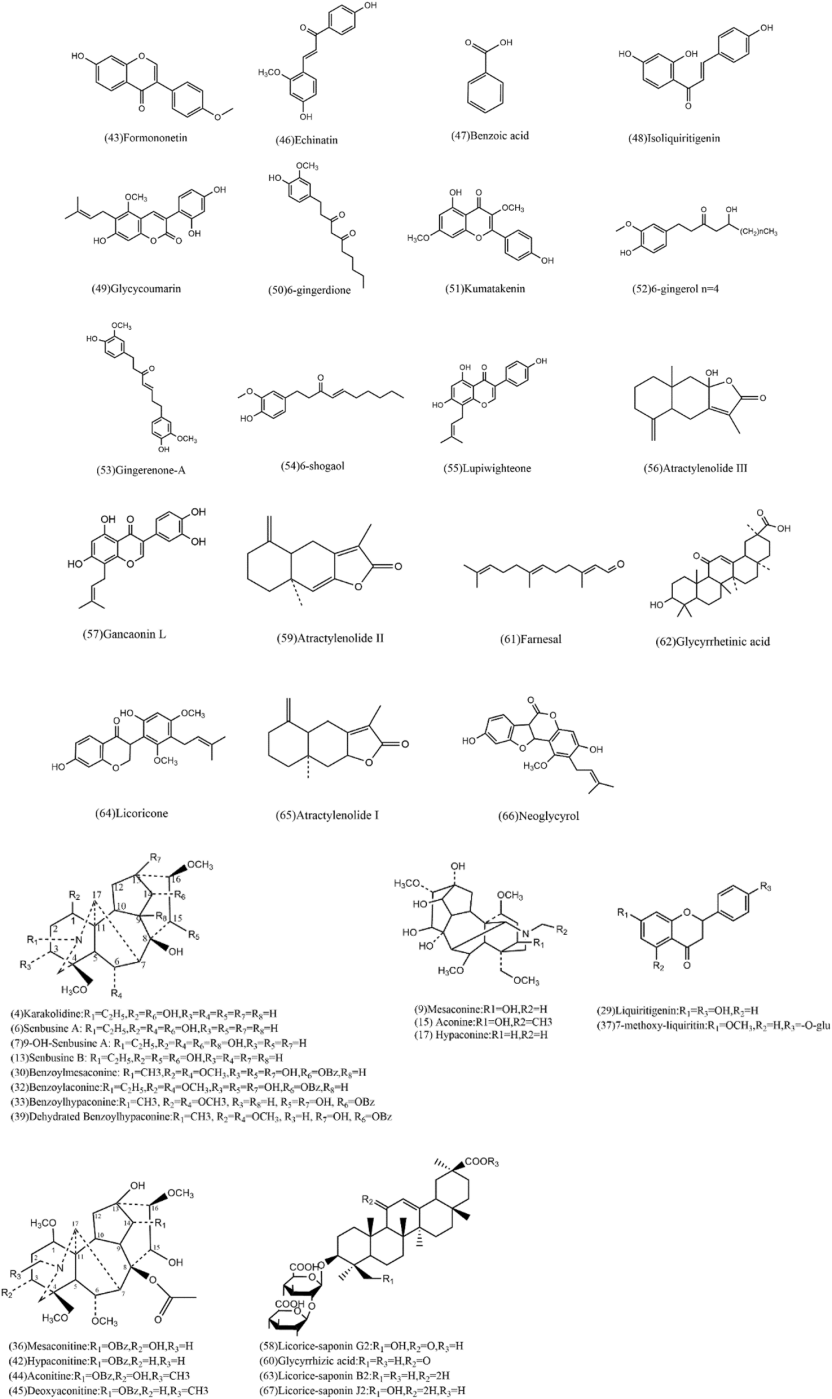
Structures of compounds identified in the extract of Fuzi Lizhong Pill

The remaining 58 compounds were tentatively characterized based on their chromatographic and spectrometric data, referring to previous literature [25, 30–33]. For example, MS^2^ spectra of compound 4 (molecular ion at *m/z* [M + H]^+^ 394.2590) in Table 2 gave characteristic fragment ions of [M + H–H_2_O]^+^ at *m/z* 376.2489 and [M + H–2H_2_O]^+^ at *m/z* 358.2371. Thus, it corresponded to Karakolidine by comparison with literature data [30]. Moreover, MS^2^ spectra of compound 12 (molecular ion at *m/z* [M + H]^+^ 408.2743) in Table 2 gave characteristic fragment ions of [M + H–H_2_O]^+^ at *m/z* 390.2630, 372.2517[M + H–2H_2_O]^+^ and [M + H–CH_3_OH]^+^ at *m/z* 358.2374. Then it was identified as Isotalatizidine. All the MS data of the (+) ESI–MS spectra are shown in Table 2. Besides, all the structures of the compounds identified are shown in Figs. 3 and 4. The deriving herb for each compound was also assigned. The majority of constituents are identified as alkaloids, flavonoids, triterpenes, gingerols, phenylpropanoids and volatile oil.

### Characterization of the absorbed chemical constituents in rat serum

#### Identification of the bioactive chemical prototype constituents in rat serum

As the results of constituents in rat serum show in Table 3, by comparing the *t*_*R*_ values and MS fragment characteristics between compounds in serum and compounds in FZLZP extract, 10 alkaloid components sourced from *Aconitum carmichaeli Debx.* were identified, including benzoylaconine, benzoylmesaconine, benzoylhypaconine, mesaconitine, Hypaconitine, fuziline, neoline, talatisamine, chasmanine, and 14-acetyltalatizamine. These constituents have been reported as parts of the main constituents with significant effects of analgesia, anti-inflammation, thermogenesis and increasing blood oxygen in Fuzi [34, 35]. The MS data of the (+) ESI–MS spectra are shown in Table 3. For example, MS^2^ spectra of compound 19 in Table 2 was detected at the Rt in 16.070 min with the molecular ion at *m/z* 454.2800[M + H]^+^ and gave characteristic fragment ions of [M + H–H_2_O]^+^ at *m/z* 436.2677. Similarly, MS^2^ spectra of compound 2 in Table 3 was detected at the Rt in 16.615 min with the molecular ion at *m/z* 454.2808[M + H]^+^ and gave characteristic fragment ions of [M + H–H_2_O]^+^ at *m/z* 436.0243. Thus, compound 2 in Table 3 was identified as the absorbed prototype of Fuziline in rat serum. The other alkaloid components were identified in a similar way.

**Table 3:** Characterization of chemical constituents in vivo and metabolites of FZLZP by HPLC-ESI/QTOF/MS

Peak no.	Rt (min)	Systematic name	Molecular formula	Molecular weight (Da)	[M + H]^+^		[M + Na]^+^		Fragmentations (m/z)	Source/prototype
					Measured value (Da)	Error (ppm)	Measured value (Da)	Error (ppm)		
1	4.841	l-Pyroglutamic acid	C_5_H_7_NO_3_	129.0426	130.0498	− 0.7689			130.0498[M + H]^+^, 112.9741[M + H–H_2_O]^+^	Dangshen
2	16.615	Fuziline	C_24_H_39_NO_7_	453.2727	454.2808	1.981			454.2808[M + H]^+^, 436.0243[M + H–H_2_O]^+^	Fuzi
3	17.021	Talatisamine	C_24_H_39_NO_5_	421.2828	422.2905	0.9472			422.2905[M + H]^+^, 390.2651[M + H–CH_3_OH]^+^, 359.3263[M + H–CH_2_OH–CH_3_OH]^+^	Fuzi
4*	24.357	Glucuronide conjugation metabolite	C_21_H_20_O_10_	432.1056	433.1132	0.6927			433.1132[M + H]^+^, 257.0843[M + H–(GluA–H_2_O)]^+^	Liquiritigenin
5	25.811	Liquiritigenin	C_15_H_12_O_4_	256.0736	257.0819	4.2788			257.0819 [M + H]^+^, 239.0713[M + H–H_2_O]^+^, 137.0237[C_7_H_4_O_3_ + H]^+^	Gancao
6	27.236	Benzoylmesaconine	C_31_H_43_NO_10_	589.2887	590.2948	− 2.033			590.2948[M + H]^+^, 558.2657[M + H–CH_3_OH]^+^ 540.2537[M + H–CH_3_OH–H_2_O]^+^, 508.2218[M + H-2CH_3_OH–H_2_O]^+^	Fuzi
7	27.520	Benzoylaconine	C_32_H_45_NO_10_	603.3043	604.3134	2.97			604.3134[M + H]^+^, 540.6158[M + H-2CH_3_OH]^+^, 508.8095[M + H-3CH_3_OH]^+^	Fuzi
8	28.379	Liquiritin or Isoliquiritin	C_21_H_22_O_9_	418.1264			441.1144	− 2.72	441.1144 [M + Na]^+^, 424.0979 [M + Na–OH]^+^, 350.8191[M + Na–C_6_H_3_O]^+^	Gancao
9	28.595	Benzoylhypaconine	C_31_H_43_NO_9_	573.2938	574.3025	0	–	–	574.3025[M + H]^+^, 443.8613[M + H-3CH_3_OH–H_2_O–HO^−^]^+^	Fuzi
10	31.405	Mesaconitine	C_33_H_45_NO_11_	631.2993	632.3079	2.2141			632.3079[M + H]^+^, 599.9372[M + H–CH_3_OH]^+^, 540.2653[M + H–AcOH–CH_3_OH]^+^	Fuzi
11	32.453	Hypaconitine	C_33_H_45_NO_10_	615.3043	616.3089	− 4.381			616.3089[M + H]^+^, 597.8211 [M + H–H_2_O]^+^, 556.2792[M + H–C_2_H_4_O_2_]^+^	Fuzi
12*	33.299	Glucuronide conjugation metabolite	C30H47NO13	629.3047	630.3295	27.7640			630.3295 [M + H]^+^, 454.8397[M + H–(GluA–H_2_O)]^+^	Fuziline
13*	33.165	Glucuronide conjugation metabolite	C_21_H_20_O_10_	432.1056	433.1145	3.6942			433.1145[M + H]^+^, 257.0829[M + H–(GluA–H_2_O)]^+^	Isoliquiritigenin
14	40.710	Isoliquiritigenin	C_15_H_12_O_4_	256.0736	257.0807	− 0.3889			257.0807[M + H]^+^, 239.1624[M + H–H_2_O]^+^	Gancao
15	42.275	6-gingerdione	C_17_H_24_O_4_	292.1675	293.1734	− 4.4342			293.1734[M + H]^+^, 275.1586[M + H–H_2_O]^+^	Ganjiang
16	42.514	Formononetin	C_16_H_12_O_4_	268.0736	269.0799	− 3.3447			269.0799[M + H]^+^, 181.0511[M + H–C_2_O_2_–CH_3_OH]^+^	Gancao
17	44.584	14-Acetyltalatizamine	C_26_H_41_NO_6_	463.2934	464.3015	1.7230			464.3015[M + H]^+^, 446.2652[M + H– H_2_O]^+^, 432.6414[M + H–CH_3_OH]^+^	Fuzi
18	46.555	6-gingerol	C_17_H_26_O_4_	294.1831	295.1905	0.3388			295.1905[M + H]^+^, 263.1618[M + H–CH_3_OH]^+^, 179.1028[M + H–C_7_H_15_O]^+^	Ganjiang
19	46.980	6-shogaol	C_17_H_24_O_3_	276.1725	277.1781	− 6.1332			277.1794[M + H]^+^, 260.1816[M + Na–OH]^+^, 245.1533[M + H–CH_3_OH]^+^	Ganjiang
20	47.690	Atractylenolide II	C_15_H_20_O_2_	232.1463	233.1533	− 1.2867			233.1533[M + Na]^+^, 187.1487[M + Na–CH_2_O_2_]^+^, 159.1179[M + Na–CH_2_O_2_–C_2_H_4_]^+^, 145.1005[M + Na–CH_2_O_2_–C_3_H_6_]^+^	Baizhu
21	48.102	Chasmanine	C_25_H_41_NO_6_	451.2934			474.2841	3.1627	474.2841[M + H]^+^, 442.0836[M + H–CH_3_OH]^+^	Fuzi
22	49.895	Glycyrrhizic acid	C_42_H_62_O_16_	822.4038	823.4094	− 2.0646			823.4094[M + H]^+^, 647.3792[M + H–(GluA–H_2_O)]^+^	Gancao
23	50.826	Atractylenolide I	C_15_H_18_O_2_	230.1307	231.1382	0.8653			231.1382[M + H]^+^, 105.9823[M + H–HCOOH–2C_2_H_4_–2C]^+^	Baizhu
24	51.095	Neoline	C_24_H_39_NO_6_	437.2777			460.2669	− 0.2173	460.2669 [M + Na]^+^, 442.2666[M + Na–H_2_O]^+^	Fuzi
25	54.144	7-hydroxycoumarin	C_9_H_6_O_3_	162.0317	163.0396	3.6801			163.0396[M + H]^+^, 145.5012[M + H–H_2_O]^+^	Baizhu
26	56.004	Glycyrrhetinic acid	C_30_H_46_O_4_	470.3396	471.3479	− 2.122			471.3479[M + H]^+^, 453.4285[M + H–H_2_O]^+^	Gancao

* Indicates metabolites

Six compounds sourced from *Glycyrrhiza uralensis* Fisch. were identified, including 3 flavonoids, namely, liquiritigenin, isoliquiritigenin, and formononetin and 2 triterpenes, namely, glycyrrhetinic acid and glycyrrhizic acid. The MS data of the (+)ESI–MS spectra are shown in Table 3. For example, MS^2^ spectra of compound 48 in Table 2 was detected at the Rt in 39.763 min with the molecular ion at *m/z* 257.0814[M + H]^+^ and gave characteristic fragment ions of 239.0704[M + H–H_2_O]^+^, 137.0235[C_7_H_4_O_3_ + H]^+^, 121.0277[C_8_H_8_O + H]^+^, 120.0527 [C_7_H_4_O_3_ + H–OH]^+^. Similarly, MS^2^ spectra of compound 14 in Table 3 was detected at the Rt in 40.710 min with the molecular ion at *m/z* 257.0807[M + H]^+^ and gave characteristic fragment ions of [M + H–H_2_O]^+^ at *m/z* 239.1624. Thus, compound 14 in Table 3 was identified as the absorbed prototype of Isoliquiritigenin in rat serum. Furthermore, liquiritin or isoliquiritin may also have been found, but further comparison with reference compounds is needed to identify these isomers. The flavonoids and triterpenes in *Glycyrrhiza uralensis* Fisch. have been reported as having significant anti-inflammatory, abirritation and immunoregulation effects [36–38].

7-Hydroxycoumarin, atractylenolide I and atractylenolide II have been identified as bioactive chemical constituents sourced form *Atractylodes macrocephala* Koidz. (Baizhu) and were found as the main institutes with the effect of anti-inflammatory, antitumor and gastrointestinal regulation in Baizhu [39–42]. The MS data of the (+) ESI–MS spectra are shown in Table 3. For example, MS^2^ spectra of compound 26 in Table 2 was detected with the molecular ion at *m/z* 163.0395 [M + H]^+^ and gave characteristic fragment ions of 145.0627[M + H–H_2_O]^+^. Similarly, MS^2^ spectra of compound 25 in Table 3 was detected with the molecular ion at *m/z* 163.0396[M + H]^+^ and gave characteristic fragment ions of [M + H–H_2_O]^+^ at *m/z* 145.5012. Thus, compound 25 in Table 3 was identified as the absorbed prototype of 7-hydroxycoumarin in rat serum.

6-Gingerdione, 6-gingerol and 6-shogaol sourced from *Zingiber officinale* Rosc (Ganjiang) were identified and were reported as having obvious antioxidant, anti-inflammatory, gastrointestinal protective and antitumor effects [43, 44]. The MS data of the (+) ESI–MS spectra are shown in Table 3. For example, MS^2^ spectra of compound 50 in Table 2 was detected with the molecular ion at *m/z* 293.1736[M + H]^+^ and gave characteristic fragment ions of 275.1650[M + H–H_2_O]^+^, 257.1517[M + H–2H_2_O]^+^. Similarly, MS^2^ spectra of compound 15 in Table 3 was detected with the molecular ion at *m/z* 293.1734[M + H]^+^ and gave characteristic fragment ions of [M + H–H_2_O]^+^ at *m/z* 257.1586. Thus, compound 15 in Table 3 was identified as the absorbed prototype of 6-gingerdione in rat serum.

One compound was sourced from *Codonopsis pilosula* (Franch.) Nannf. (Dangshen) and was identified as l-pyroglutamic acid. MS^2^ spectra of compound 1 in Table 2 was detected with the molecular ion at *m/z* 130.0505[M + H]^+^ and gave characteristic fragment ions of 112.0123[M + H–H_2_O]^+^, 84.0449[M + H–HCOOH]^+^. Similarly, MS^2^ spectra of compound 1 in Table 3 was detected with the molecular ion at *m/z* 130.0498[M + H]^+^ and gave characteristic fragment ions of [M + H–H_2_O]^+^ at *m/z* 112.9741. Thus, compound 1 in Table 3 was identified as the absorbed prototype of l-pyroglutamic acid in rat serum.

#### Identification of the bioactive metabolites in rat serum

Based on a comparison of the information for ions, 8 peaks were detected only in dosed serum and were assigned to metabolites. Detailed information about the elemental compositions, retention times, and the characteristic fragment ions of metabolites are shown in Table 3. Alkaloid-, phenylpropanoids- and gingerols-related metabolites are the main metabolic constituents of FZPLP absorbed in vivo, and the main metabolic pathways in vivo were glucuronide conjugation and glucuronide. Identification of the corresponding fragment ions was obvious. For example, compound 4 (24.357 min) in Table 3 produced [M + H] + at *m/z* 433 and MS^2^ yielded a major ion at *m/z* 257 (− 176, Da with the loss of C_6_H_8_O_6_) in the positive ion mode, combined with the retention time of the reference standard 1 in Table 1 and compound 29 in Table 2. Therefore, the peak was identified tentatively as a glucuronide conjugation metabolite of liquiritigenin. Similarly, compound 13 (the *t*_*R*_ 33.165 min) in Table 3 has the similar retention time compared with the reference standard 6 in Table 1 and compound 48 in Table 2. And it produced [M + H] + at *m/z* 433 and MS^2^ yielded a major ion at *m/z* 257 (− 176, Da with the loss of C_6_H_8_O_6_) in the positive ion mode. Therefore, the peak was identified tentatively as a glucuronide conjugation metabolite of isoliquiritigenin. The possible structures of metabolites were elucidated as described above. All of the structures of metabolites were identified, and the MS data of the (+) ESI–MS spectra are shown in Table 3. This article reports these metabolites of FZLZP for the first time. The bioactivities are the subject of ongoing research.

### Alkaloids difference between Group A and Group B

**Fig. 5:**
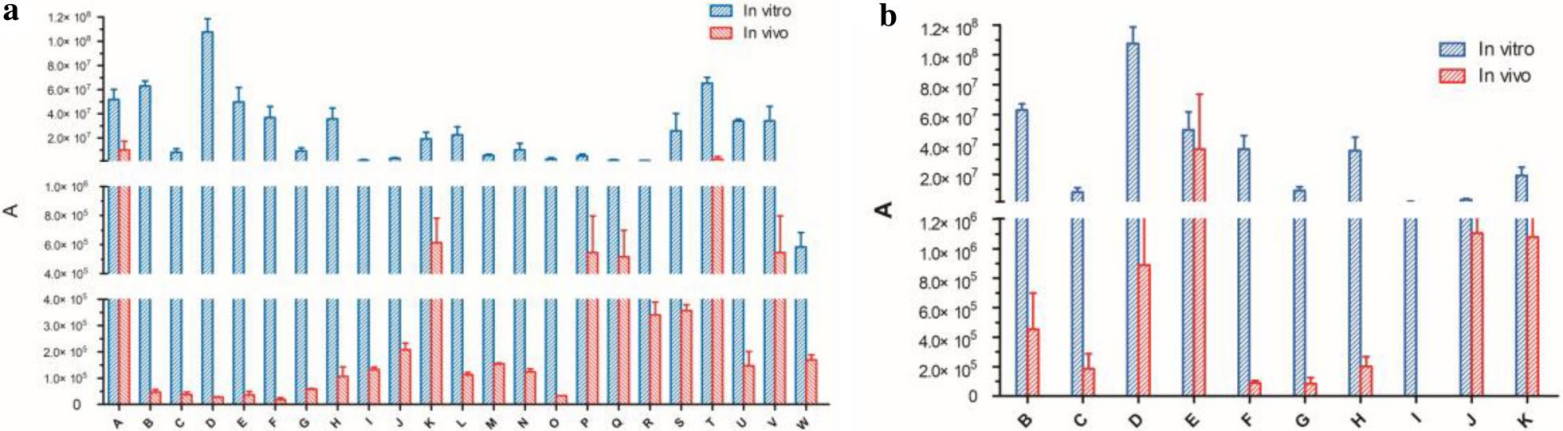
The difference in the absorbed compounds in vitro and in vivo. (**a** The difference in the absorbed compounds in vitro and in vivo of Group A; **b** The difference in the absorbed alkaloids in vitro and in vivo of Group B.) (Columns: A, l-pyroglutamic acid; B, Fuziline; C, Talatisamine; D, Benzoylmesaconine; E, Benzoylaconine; F, Benzoylhypaconine; G, Mesaconitine; H, Hypaconitine; I, 14-Acetyltalatizamine; J, Chasmanine; K, Neoline; L, Liquiritigenin; M, Liquiritin or Isoliquiritin; N, Isoliquiritigenin; O, Formononetin; P, Glycyrrhizic acid; Q, Glycyrrhetinic acid; R, 6-gingerdione; S, 6-gingerol; T, 6-shogaol; U, Atractylenolide II; V, Atractylenolide I; W, 7-hydroxycoumarin)

As the result shows in Fig. 5a, 10 kinds of alkaloids were detected in Group A. Most of them were trace amounts in vivo, which indicated the alkaloids’ poor absorption in the prescription. Conversely, unlike Group A, the amount of the alkaloids in vivo increased obviously in Group B (Fig. 5b). The difference indicated that the absorption amount of alkaloids in the prescription can be decreased compared to the absorption amount of alkaloids in the herb powder.

## Discussion

To obtain LC chromatograms of lower pressure, greater baseline stability, better resolution and higher ionization efficiency, methanol and acetonitrile and series of concentrations of aqueous formic acid solution were prepared for analysis. The best result was achieved when the mobile phase consisted of 0.1% formic acid aqueous solution and methanol. Both positive and negative modes were investigated, and the results showed that the positive ion mode was more sensitive and could provide more information for both extract samples and serum samples analyses.

FZLZP is a formula composed under the guidance of traditional Chinese medicine theory. According to TCM theory, *Aconitum carmichaeli Debx.* is the “monarch drug” and the main herb in FZLZP recipe to warm middle jiao and eliminate cold. This was confirmed in this research with 10 constituents among 23 prototype components sourced from *Aconitum carmichaeli* Debx., which maintains the maximum bioactive compounds. *Glycyrrhiza uralensis* Fisch. is frequently prescribed in combination with other herbs to decrease toxicity and to increase efficacy. In this recipe, it is the “envoy drug” and is considered to be the paramount assistant herb, which can detoxify the toxicity of aconitum. In this study, we found that *Glycyrrhiza uralensis* Fisch. was the second most-absorbed herb. The results that some compounds absorbed well in vivo derived from *Aconitum carmichaeli Debx.* and *Glycyrrhiza uralensis* Fisch. are consistent with our previous studies that they were dissolved very well in vitro [16].

Alkaloids in Fuzi herb are the toxicity as well as the efficacy compounds. The prescriptions which contains Fuzi herb should be highly concerned. In our study, the results on the differences in alkaloids between Group A and Group B show that the amount of absorption of bioactive constituents in Fuzi can be significantly reduced when this herb is used as part of a prescription rather than used alone. We think there are two reasons. Firstly, according to the TCM theory, the toxicity of Fuzi can be reduced in combination with Gancao [25]. This should be further confirmed by researching the relationship and differences in the chemistry constituents between Fuzi-Gancao herb pairs in FZLZP. Secondly, the pill form is the embryonic form of sustained-release preparations. As a TCM classic says: only pill among all dosage forms can reduce the toxicity of toxic drugs. The toxic herb was usually made into a pill form to reduce the toxicity in TCM [17]. And it can be further confirmed by researching differences in the chemistry constituents between FZLZP and the Fuzi pill that made from* Aconitum carmichaeli Debx*. powder.

## Conclusions

This study describes a simple, sensitive and selective HPLC-QTOF-MS method for structural characterization of chemical constituents in FZLZP and bioactive components in rat serum following oral administration of FZLZP. As a result, in vitro, a total of 67 compounds were successfully identified, and 23 prototype compounds that were absorbed in vivo were identified for the first time. In addition, 3 metabolites of the bioactive compounds were tentatively identified. In this prescription, the majority of compounds absorbed in vivo derived from Fuzi and Gancao. The results provide helpful chemical information for FZLZP for further pharmacological and active mechanism research. In addition, it helped to classify the material basis responsible for the therapeutic effects of FZLZP. Furthermore, the HPLC-QTOF-MS was a potentially powerful strategy for simultaneously achieving screening and analysis of multiple bioactive compounds in FZLZP.

## Additional file

**Additional file 1.** Minimum standards of reporting checklist.

## Abbreviations

HPLC-QTOF-MS: high-performance liquid chromatography–electrospray ionization/quadrupole-time-of-flight high-definition mass spectrometry
FZLZP: Fuzi Lizhong Pill
FZP: Fuzi powder
Group A: FZLZP group for dosed rat serum
Group B: Fuzi powder group for dosed rat serum
Group C: control group for blank rat serum
